# Neuropathic Pain: Delving into the Oxidative Origin and the Possible Implication of Transient Receptor Potential Channels

**DOI:** 10.3389/fphys.2018.00095

**Published:** 2018-02-14

**Authors:** Cristina Carrasco, Mustafa Naziroǧlu, Ana B. Rodríguez, José A. Pariente

**Affiliations:** ^1^Department of Physiology, Faculty of Sciences, University of Extremadura, Badajoz, Spain; ^2^Neuroscience Research Center, Suleyman Demirel University, Isparta, Turkey

**Keywords:** neuropathic pain, oxidative stress, inflammation, mitochondrial dysfunction, TRP channels, antioxidants

## Abstract

Currently, neuropathic pain is an underestimated socioeconomic health problem affecting millions of people worldwide, which incidence may increase in the next years due to chronification of several diseases, such as cancer and diabetes. Growing evidence links neuropathic pain present in several disorders [i.e., spinal cord injury (SCI), cancer, diabetes and alcoholism] to central sensitization, as a global result of mitochondrial dysfunction induced by oxidative and nitrosative stress. Additionally, inflammatory signals and the overload in intracellular calcium ion could be also implicated in this complex network that has not yet been elucidated. Recently, calcium channels namely transient receptor potential (TRP) superfamily, including members of the subfamilies A (TRAP1), M (TRPM2 and 7), and V (TRPV1 and 4), have demonstrated to play a role in the nociception mediated by sensory neurons. Therefore, as neuropathic pain could be a consequence of the imbalance between reactive oxygen species and endogen antioxidants, antioxidant supplementation may be a treatment option. This kind of therapy would exert its beneficial action through antioxidant and immunoregulatory functions, optimizing mitochondrial function and even increasing the biogenesis of this vital organelle; on balance, antioxidant supplementation would improve the patient's quality of life. This review seeks to deepen on current knowledge about neuropathic pain, summarizing clinical conditions and probable causes, the relationship existing between oxidative stress, mitochondrial dysfunction and TRP channels activation, and scientific evidence related to antioxidant supplementation.

## Introduction

Currently, neuropathic pain (NP) is an underestimated socioeconomic health problem affecting millions of people worldwide. It has been recently redefined by the International Association for the Study of Pain as a “pain caused by lesion or disease of the somatosensory system” and it may appear in a wide range of conditions; it can be classified into peripheral or central NP, depending on anatomic location of the lesion or disease. Without a specific diagnostic tool, both clinicians and researchers might use a grading system with different levels of certainty about the presence of NP (“possible,” “probable,” and “definite”) in a patient; however, it should be mentioned that coexisting NP and other types of pains (such as nociceptive pain) can make difficult a reliable distinction (Treede et al., 2008; Jensen et al., 2011). The common symptoms of different types of NP are mechanical allodynia and hyperalgesia. Unlike nociceptive pain, commonly prescribed analgesics often fail in alleviating NP. Thus, it can become a chronic and hardly bearable condition, recently called refractory NP. In some cases, this most severe NP may lead to increased episodes of depression and suicide (Torrance et al., 2013; Hassler et al., 2014; Kawaguchi et al., 2014).

**Figure 1:**
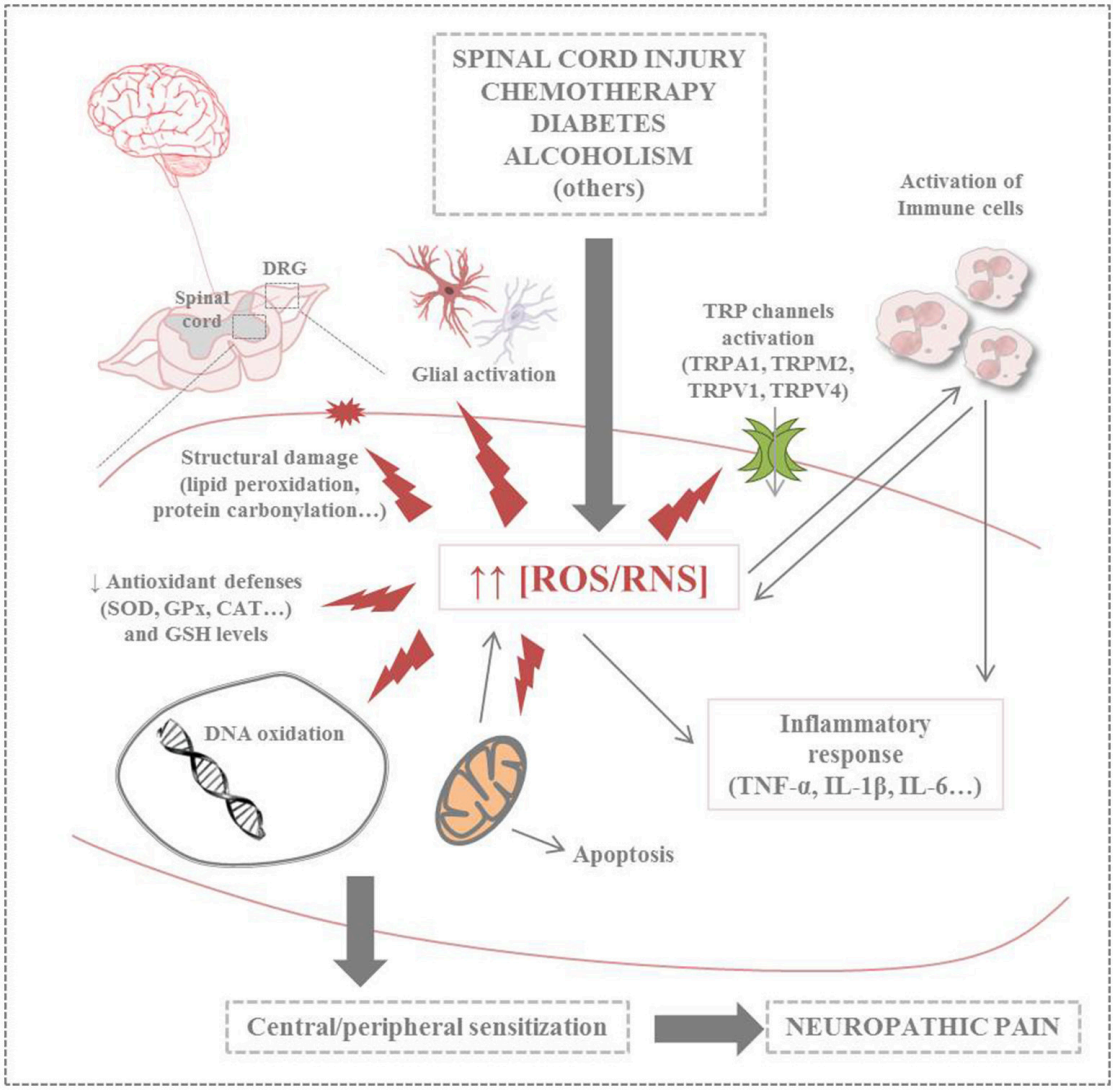
Summary of harmful effects of nitro-oxidative stress on neuronal cells in neuropathic pain (CAT, catalase; GPx, glutathione peroxidase; GSH, glutathione; IL-1/6, interleukin 1/6; ROS/RNS, reactive oxygen species/reactive nitrogen species; SOD, superoxide dismutase; TNF-α, tumor necrosis factor-α; TRP, transient receptor potential; TRPA1, transient receptor potential ankyrin 1; TRPM2, transient receptor potential melastatin 2; TRPV1/4, transient receptor potential vanilloid 1/4).

Further research is needed to understand underlying mechanisms of NP that allow to design individual and rational treatment strategies. Growing evidence points that mitochondrial dysfunction induced by oxidative and nitrosative stress, along with inflammation, constitute the physiopathological basis for the development of several diseases (Carrasco et al., 2015). Regarding to NP, this adverse context leads to peripheral and central sensitization. It must be considered that mammalian nerves are especially susceptible to free radicals, including oxygen (ROS) and nitrogen reactive species (RNS), due to their high content in phospholipids and axonal mitochondrion; in addition, neuronal antioxidant defenses are weak (Areti et al., 2014). Studies about antioxidant supplementation in animal models of NP point that hydroxyl (^•^OH) and superoxide (${\text{O}}_{2}^{•-}$) radicals and nitric oxide (^•^NO) might be involved in the physiopathology of this kind of pain (Kawaguchi et al., 2014). However, the way these messenger molecules regulate pain signaling is still poorly understood. In addition, overload intracellular calcium ion (Ca^2+^) has also an important role in the etiology of NP. Ca^2+^ enters cells in different ways including cation channels. Voltage gated calcium channels and chemical channels (i.e., glutamate) are well known calcium channels (Kumar et al., 2014). Moreover, new calcium channels namely transient receptor potential (TRP) superfamily were discovered in eye cells of Drosophila flyers (Hardie, 2011; Naziroǧlu, 2011). In different species, TRP superfamily is divided into 30 channels within seven subfamilies such as TRPA (ankyrin), TRPC (canonical), TRPM (melastatin), TRPML (mucolipin), TRPP (polycystin), TRPV (vanilloid), and TPRN (NomPC), nevertheless there are 28 subfamilies within 6 subgroups in mammalian (Hardie, 2011; Naziroǧlu, 2011; Uchida et al., 2017). At least, nine members of TRP superfamily are activated by oxidative stress including TRPM2, TRPM7, TRPA1, TRPC3, TRPC5, TRPC6, TRPV1, TRPV3, and TRPV4 (Ogawa et al., 2016). Dorsal root ganglion (DRG) neurons play an important role in the painful NP. There is no barrier between the DRG and blood and compounds with high molecular weight can easily diffuse into the DRG (Abram et al., 2006). Expression levels of TRPA1, TRPM2, TRPV1, and TRPV4 channels are high in the DRG and trigeminal ganglia neurons (Kobayashi et al., 2005; Obata et al., 2005; Fonfria et al., 2006; Naziroǧlu, 2011). Hence, the TRPA1, TRPM2, TRPV1, and TRPV4 play an important role in the nociception mediated by sensory neurons, including the DRG (Materazzi et al., 2012; Özdemir et al., 2016; Kahya et al., 2017). Therefore, increased ROS/RNS levels induced by several clinical conditions may play a deleterious effect on different biomolecules (e.g., lipids, proteins and nucleic acids), organelles and antioxidant defenses, leading to exacerbate nitro-oxidative stress, mitochondrial dysfunction, glial activation and inflammatory response. Recent evidence also points out the possible implication of TRP channels in NP. Altogether, this adverse context is ultimately responsible of the typical painful symptoms of NP (Figure 1).

This review summarizes current knowledge about NP, focusing on clinical conditions and probable causes, the relationship existing among oxidative stress, mitochondrial dysfunction and TRP channels activation and scientific evidence related to antioxidant supplementation.

### Spinal cord injury

Following spinal cord injury (SCI), individuals suffer not only motor dysfunction but also the development of chronic NP. Up to 80% of patients experience this condition within months after injury, which dramatically impairs their quality of life; thus, taking in account that this kind of NP is refractory to clinical treatments, depression and suicide are very frequently (Stillman et al., 2017). It is thought that after SCI, many neuroadaptation responses are implemented in dorsal horn, triggering central mechanisms which likely contribute to NP. Apart from dysfunction of neurons, other pathogenic events have been defined about post-SCI pain, including pro-inflammatory signaling, microglia activation and intracellular Ca^2+^ alteration; however, little is known about how oxidative stress may play a critical role in this condition (Hulsebosch et al., 2009; Due et al., 2014).

Evidence supports that after a central nervous system injury, increased extracellular glutamate levels activate several intracellular pathways including ROS formation; this change in redox status promotes a leukocyte mediated pro-inflammatory response that ultimately leads to the exacerbation of secondary damage (Hulsebosch et al., 2009). In this sense, some studies have implicated the lipid peroxidation byproduct (acrolein) in many neuropathological diseases and NP modalities (Shi et al., 2011). During inflammation and trauma acrolein is released, causing damage to biomolecules and altering several cellular processes in neurons, including mitochondria functionality. Consequently, this aldehyde is well known to be a potent oxidant, perpetuating a vicious cycle of oxidative stress which may partially explain its role as TRPA1 agonist. Due et al. (2014) have reported increased levels of acrolein within or near the injured site for at least 2 weeks following experimental SCI, a phenomenon which was correlated with the onset of sensory and behavioral hypersensitivity in rats. Moreover, exogenous administration of acrolein into rodent spinal cord also induced pain symptomatology, whereas the treatment with its scavenger hydralazine modestly diminished sensitivity to both tactile and thermal stimuli. Researchers also performed *in vitro* assays observing that acrolein increases neuronal excitation. Together, these findings strongly support the pro-nociceptive role of acrolein likely via TRPA1 activation, as deduced by increased levels of this receptor in sensory ganglia also observed in the referred study. But as authors mentioned, other TRP channels might also be sensitive to acrolein and contribute to SCI-induced NP. In accordance with these observations, Park et al. (2014) have also reported *in vivo* evidence about the crucial role of acrolein in the pathogenesis of spinal cord trauma and the potential use of hydralazine as analgesic. In this study, a significant reduction of acrolein levels, tissue damage, motor deficits and NP was observed in hydralazine-treated rats.

On the other hand, other mediators may also be part of the pathophysiological mechanisms triggered by oxidative stress in this kind of NP, such as aquaporin-1. Apart from mediating several physiological processes via water transport, aquaporin-1 must also play a not still well understood role in the etiology of different neuropathological conditions; it is supposed to contribute to some of the typical outcomes such as edema and cyst formation. In experimental models of SCI, this protein has been found to be significantly elevated for up to 11 months in sensory axons, neurons, astrocytes, and ependymal cells, despite consequent loss of nerve tissue at the site of injury. Experimental data point oxidative stress as one of the factors that contributes to aquaporin-1 up-regulation since administration of the antioxidant melatonin not only reduced protein levels but also mechanical allodynia and appearance of aquaporin-1-positive fibers below laminae I and II (Nesic et al., 2008).

More recently, *in vivo* proteomic approaches of SCI have demonstrated that peripheral nerve injury alters the expression and/or subcellular distribution of some specific dorsal horn proteins, for example those involved in nociceptive signaling, cellular metabolism, plasma membrane receptor trafficking, oxidative stress, apoptosis and degeneration (Lee et al., 2003; Kunz et al., 2005; Singh et al., 2009). In this sense, the TRP superfamily's member TRPM4 should be mentioned. TRPM4 is a non-selective, Ca^2+^-impermeable channel that exclusively transports monovalent cations. TRPM4 is activated by increased intracellular ATP concentration and oxidative stress, but it is inhibited by intracellular ATP depletion (Nilius et al., 2004; Simon et al., 2010). Involvement of the Cys1093 residue oxidation in TRPM4 channel activation has also a significant role in oxidative stress dependent (without ATP depletion) activation and desensitization of TRPM4. Involvement of hydrogen peroxide (H_2_O_2_) is well known for induction of necrosis; in endogenously expressed TRPM4 HeLa cells, H_2_O_2_ induction of both necrosis and apoptosis has been shown (Simon et al., 2010). TRPM4 has an important role for induction of neurological diseases. For example, involvement of TRPM4 in etiology of SCI was reported by result of recent papers (Gerzanich et al., 2009; Lee et al., 2014).

### Chemotherapy-induced peripheral neuropathy

Many first-line chemotherapy agents used in the current clinical practice, such as platinum-based anticancer drugs (i.e., cisplatin, oxaliplatin), proteasome/angiogenesis inhibitors (bortezomib/thalidomide), vinca alkaloids (i.e., vincristine, vinorelbine) and taxanes (i.e., paclitaxel, docetaxel) cause a dose-limiting side effect called chemotherapy-induced peripheral neuropathy (CIPN) (Han and Smith, 2013; Kerckhove et al., 2017). This kind of NP involves predominantly sensory nerves and occurs in a stocking-and-glove distribution. Depending on anticancer drugs, 38–100% of cancer patients are affected by CIPN and its symptoms (mainly allodynia and hypersensitivity) may persist from months to years following cessation of anticancer treatment, a phenomenon known as *coasting*. It seems that chemotherapy regimens (drug or combination of drugs administered and dosing), methods of pain assessment and the individual patient characteristics (presence of comorbidities associated with increased risk of neuropathy, such as diabetes, depression, insomnia or genetic particularities) are some of the several factors that may influence on the onset of CIPN and the severity of symptoms. In addition, most of the effective analgesics in NP failed to provide symptomatic relief of CIPN and often exhibit side effects (Han and Smith, 2013; Griffiths and Flatters, 2015). Thus, CIPN may limit the dosing, duration and effectiveness of the treatment, affecting survival and quality of life of the patient (Ji et al., 2013; Kerckhove et al., 2017).

In general, unlike other types of NP (including those induced by trauma and diabetes) axonal degeneration in peripheral nerves is not present in CIPN (Ji et al., 2013). For this reason, NP is suspected to be a complex phenomenon resulting from the interrelation of various mechanisms. It has been observed that anticancer drugs may cause neuronal damage in a variety of ways, such as nuclear and mitochondrial DNA damage, ion channel disturbances (i.e., calcium, sodium and potassium), impairment of axonal transport and inflammatory process (Han and Smith, 2013; Massicot et al., 2013; Kerckhove et al., 2017). More recently, experimental evidence points oxidative stress and mitochondrial dysfunction as one of the common pathophysiological mechanisms responsible of neurotoxicity in CIPN. Thus, overproduction of ROS and RNS may affect redox status toward oxidation, interfering with the antioxidant defense system and cell function. An increase in several markers of oxidative stress (lipid peroxidation, carbonylated proteins and DNA oxidation) in experimental models of CIPN has been observed; moreover, homozygous individuals for GSTP1 105Ile allele, that encode the oxidative stress regulatory enzyme glutathione S-transferase pi 1, tend to suffer this type of NP more frequently than other people (Han and Smith, 2013). At mitochondrial level, structural integrity and energy function of this organelle are affected, triggering the apoptosis cascade. Furthermore, inefficient autophagy/mitophagy of damaged biomolecules and organelles leads to a vicious cycle in the cells, which ultimately exacerbates the progression of the CIPN typical neurodegeneration. Consequently, inflammatory response is activated in both neurons and immune system cells (Massicot et al., 2013; Areti et al., 2014). Specific oxidative damage features in CIPN induced by most commonly used chemotherapeutic agents are detailed below.

Regarding to CIPN induced by the taxanes, Duggett et al. (2016) analyzed oxidative stress in DRG neurons of rats treated with paclitaxel at three time-points (prior to pain onset (day 7), during (peak pain) and at resolution of pain). Whereas researchers did not find any change in mitochondrial ROS or ${\text{O}}_{2}^{•-}$ levels while studying entire neuronal populations, analysis of separately subpopulations of nociceptive neurons showed a statistically significant increase of ROS levels in isolectin B4-positive neurons from treated rats compared to control rats at the three time-points studied. For authors, these results indicate that neuronal antioxidant defenses seem to be initially overwhelmed, leading to ROS overproduction prior to pain onset; this phenomenon causes mitochondrial dysfunction with increased superoxide radical levels at peak pain, triggering signaling cascades that may underlie the coasting effect, such as apoptosis. It has been observed that the apoptotic process in paclitaxel induced-painful neuropathy is due to the permeability transition pore opening, with the consequent cytochrome c release and Ca^2+^ homeostasis dysregulation (Areti et al., 2014). In this sense, Griffiths et al. (Griffiths and Flatters, 2015) reported that the selective pharmacological modulation of the mitochondrial electron transport chain (ETC) (at level of complex I or III) *in vivo* could also reverse or attenuate the above-mentioned symptoms, but with deleterious effects on motor coordination in the case of complex I inhibition at 3 and 24 h after paclitaxel administration; however, only complex III inhibition before and during the chemotherapy exposure caused an effective relief of pain in prophylactic studies. According to authors, paclitaxel administration causes mitochondrial dysfunction as first oxidative event, resulting in excessive ROS levels which could not be counteracted by the weak antioxidant defenses of neurons; due to this fact, ROS-driven pain behaviors start. In addition, other studies have also demonstrated the involvement of TRP channels in paclitaxel-induced pain, which are well known to be predominantly expressed on this subpopulation of DRG neurons. In fact, a specific cellular signaling pathway has been described in mice, including the activation of protease-activated receptor 2 and downstream enzymes phospholipase C and protein kinases A and C by mast cell triptase, and resultant sensitization of TRPV1, TRPV4, and TRPA1 (Chen et al., 2011). Later, Materazzi et al. (2012) observed *in vitro* that paclitaxel administration induced oxidative stress byproducts which ultimately activate TRPA1 and TRPV4. Thus, pharmacological TRP channel targeting may offer a future possibility to attenuate paclitaxel-induced mechanical and thermal hypersensitivity in clinical practice. In summary, as Kerckhove et al. (2017) point the oxidative stress in taxanes induced-NP not only causes damage to neuronal and non-neuronal cells, but also macrophage activation, with the consequent overproduction of pro-inflammatory cytokines, such as TNF-α and IL-1β. In fact, Li et al. (2015) have recently reported that paclitaxel treatment may also trigger pro-inflammatory mechanisms in DRG neurons via toll-like receptor 4, which is also associated to the sensitization of TRPV1.

On the other hand, platinum compounds such as oxaliplatin have a strong neurotoxicity compared to other anticancer drugs; more than 90% of patients develop acute neuropathy which could become chronic in a 30–50% of the cases. However, the complex machinery underlying CIPN is poorly understood. To date, it has been observed that the treatment with this kind of antitumor agents causes ROS generation, mitochondrial dysfunction (including frataxin deficiency, mitochondrial DNA damage and defective components of ETC), loss in antioxidant enzymes, ion channels disturbances and nerve tissue damage (protein carbonylation and lipid peroxidation) (Areti et al., 2014; Kerckhove et al., 2017). It is thought that various receptors and molecular pathways must be involved in the sensitization of peripheral and central sensory nerves, and thus, in the chronicity of CIPN. Recently, Massicot et al. (2013) have described *in vitro* and *in vivo* biochemical effects after exposure to high concentrations of oxaliplatin, including neuronal activation of the purinoreceptor subtype 7, ROS and ^•^NO production, lipid peroxidation, loss of mitochondrial transmembrane potential and further apoptosis via caspase 3 activation. Regarding to immune response, oxaliplatin-treated neurons release significant amounts of pro-inflammatory cytokines, mainly TNF-α and IL-6, whereas prostaglandin E2 levels were significantly higher in macrophagues exposed to the platinum compound than in control cells. These inflammatory mediators might stimulate nociceptors, leading to generation and further chronification of painful symptoms. Overall, authors suggest that oxidative stress along with purinoreceptor subtype 7/inflammasome pathway would play a persistent role in oxaliplatin-induced neurotoxicity and its transition from acute to chronic NP. In addition, purinoreceptor subtype 7 activation has been observed to cause caspase-1 activation, which is ultimately involved in the expression of cyclooxygenase 2 and prostaglandin E2 and IL-β production.

Recently, vinca alkaloids have demonstrated to exert their neurotoxicity via activation of spinal cord glia (i.e., astrocytes and microglia), offering an option for the treatment of CIPN through pharmacological antagonism of this phenomenon. For authors such as Ji et al. (2013) spinal astrocytic activation, but no microglial activation, seems to contribute to mechanical allodynia in vincristine-treated rats, leading to overexpression of IL-β. This pro-inflammatory mediator might bind to its endogenous receptor to induce N-methyl-D-aspartic acid receptor phosphorylation in spinal dorsal horn neurons; thus, neuronal activity and nociceptive signaling would be enhanced. On the contrary, other studies have reported both astrocytic and microglial activation (Sweitzer et al., 2006; Kiguchi et al., 2008) after vincristine exposure, as well as up-regulation of other inflammatory cytokines such as TNF-α (Kiguchi et al., 2008). As mentioned by Ji et al. (2013), methodological differences (i.e., animal model, dosage and route of administration, etc.) might be one of the reasons for this discrepancy. In any case, oxidative stress would trigger the glial activation, as well as other processes. A study performed *in vivo* has revealed that vincristine-induced ROS overproduction may also increase the activity of the enzyme dipeptidylpeptidase IV and decrease the levels of spinal endomorphin-2. Authors' hypothesis reveals that the loss of this kind of endogenous inhibitory signal might contribute to allodynia and central sensitization, with the subsequent development of chronic pain.

Finally, bortemozib and thalidomide are well known to act as proteasome/angiogenesis inhibitors. However, a proteasome-independent mechanism might also contribute to CIPN; thus, mitochondrial dysfunction may also be involved (Kerckhove et al., 2017). Recently, Zheng et al. (2012) have reported in rat sciatic nerve how bortemozib, as other antitumor drugs, causes significant deficits in complex I and II of ETC, as well as in ATP production, at two time points (pain onset -day 7- and peak pain -day 35-).

**Figure 2:**
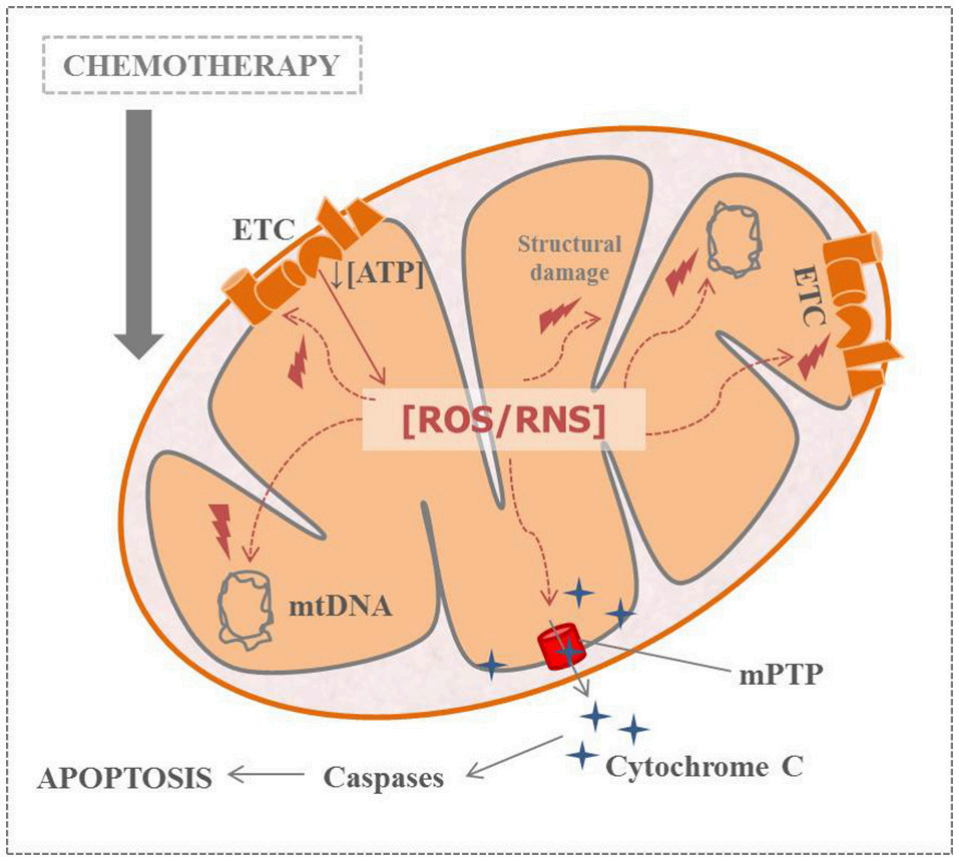
Relation between nitro-oxidative stress, mitochondrial dysfunction and apoptosis in chemotherapy-induced peripheral neuropathy (ATP, adenosine triphosphate; ETC, electronic transport channel; mtDNA, mitochondrial DNA; mPTP, mitochondrial permeability transition pore; ROS/RNS, reactive oxygen species/reactive nitrogen species).

Therefore, scientific evidence highlights the importance of early oxidative stress in the CIPN onset. But the important question that remains unresolved is: what happens first after chemotherapy administration? Mitochondrial dysfunction or increased ROS levels? In any case, this context leads to a vicious cycle which compromises ATP production and neuronal viability (Figure 2). Experts agree that, experimentally, the period between the last injection of the antitumoral agent and the time of normal onset of pain is a crucial window for an effective ROS scavenging. Taking into account all the above-mentioned, monitoring oxidative stress related-parameters during the course of CIPN could be helpful in clinical practice (Areti et al., 2014). Moreover, some studies have demonstrated the effectiveness of antioxidant therapies in this kind of neuropathic pain. These promising results about antioxidant supplementation in CIPN will be discussed later.

### Diabetic neuropathy

Peripheral diabetic neuropathy (PDN) is the most common diabetic complication in patients with diabetes (both type 1 and type 2). It may appear as a painful and insensate neuropathy, compromising patient's functionality, mood and quality of life. Symptomatology (i.e., paresthesia, spontaneous pain, tactile allodynia and mechanical and thermal hypo/hyperalgesia) may improve and resolve spontaneously, or culminate in total loss of sensation and ultimately in foot ulceration and amputation. Current clinical strategies for the management of PDN include glycolic control and treatment with drugs such as tricyclic compounds, serotonin noradrenalin reuptake inhibitors, α-lipoic acid, anticonvulsants, opiates, membrane stabilizers and topical capsaicin; however, these therapeutic options are often inefficient, have significant side effects and its prescription depends on the presence of comorbidities (Obrosova et al., 2008; Pacher, 2008; Obrosova, 2009; Tesfaye, 2011; Ma et al., 2015).

Pathogenesis of PDN is poorly understood, but it is quite clear that hyperglycemia plays a vital role in the development of this diabetic complication. Experimental evidence points a multifactorial etiology, linking hyperglycemia with the activation of multiple cell phenomena such as polyol pathway, advanced glycation end-products formation, protein kinase C (PKC) and nuclear factor Kβ signaling, among many others (Pacher, 2008; Obrosova, 2009). It should be noted that, among other physiological effects, chronic hyperglycemia leads to an imbalance of the oxidative status, affecting central and peripheral nervous system; hence, nitro-oxidative stress is also thought to be one of the responsible factors of nervous degeneration that characterize PDN (Mirshekar et al., 2010; Tesfaye, 2011). In diabetic state, hyperglycemia leads to overproduction of free radicals (i.e., mitochondrial ${\text{O}}_{2}^{•-}$, ^•^NO and peroxynitrite) mainly derived from glucose oxidation and lipid peroxidation, which cause oxidative damage to biomolecules. In particular, it is well known that oxidative DNA damage triggers the over-activation of the nuclear enzyme poly (ADP-ribose) polymerase-1 (PARP-1). An *in vivo* study performed by Obrosova (2009) demonstrated that administration of a PARP inhibitor counteract small sensory nerve fiber dysfunction and degeneration. Therefore, PARP-1 activation seems to play an important role in the pathogenesis of PDN in several ways, likely including the regulation of various important inflammatory pathways. In this sense, other studies have observed increased levels of TNF-α in diabetic animal tissues (Satoh et al., 2003; Skundric and Lisak, 2003). In addition, Ma et al. (2015) reported the role of mitochondrial bioenergetics deficits in PDN and its possible link with immune response. As authors highlighted, inflammatory signaling may lead to inhibition of ETC activity, through induction of changes in the phosphorylation state of proteins and the reduction of the mitochondrial membrane potential. Taking evidence in account, more studies are necessary to go deep into the participation of oxidative stress, mitochondrial dysfunction and inflammatory response in the development of PDN.

### Alcoholic peripheral neuropathy

Long-term excessive consumption of alcohol may lead to a condition known as alcoholic peripheral neuropathy (APN). Like other types of NP, it is characterized by spontaneous pain, hyperalgesia and allodynia. Regarding to APN related-risk factors, duration and amount of total lifetime alcohol consumption have been demonstrated as the most determinants; interestingly, a higher prevalence has been found in women than men (Chopra and Tiwari, 2012). Although little is known about physiopathological mechanisms underlying APN, a combination of direct toxic effects of ethanol or its metabolites and nutritional deficiencies (mainly thiamine) may offer a plausible explanation to this complication.

To date, different molecular mechanisms (i.e., PKC and nuclear factor Kβ) (Dina et al., 2000), signaling pathways (i.e., MEK/ERK and apoptosis via caspase activation) (Jung et al., 2005; Dina et al., 2007), receptors (i.e., metabotropic glutamate and μ opioid receptors) (Miyoshi et al., 2007; Narita et al., 2007), nerve cells (i.e., astrocytes and microglia) (Narita et al., 2007) and neuroendocrine stress axis (i.e., sympatho-adrenal and hypothalamo-pituitary-adrenal axis) (Dina et al., 2008) have shown to be involved in the APN. Focusing oxidative stress, some studies have indicated that in heavy drinkers increased nitro-oxidative stress plays a pivotal role in the neuronal damage. This imbalance in redox status might be caused by acetaldehyde, a highly toxic and reactive metabolite derived from the biphasic catabolic conversion of ethanol to acetate, in particular, as a byproduct of the mitochondrial enzyme acetaldehyde dehydrogenase. In the liver, acetaldehyde is known to cause impairment of mitochondrial ETC and stimulation of inflammatory response, among other toxic effects (Chopra and Tiwari, 2012). Therefore, mitochondrial dysfunction may also lead to an inefficient detoxification and subsequent accumulation of acetaldehyde, worsening redox status and cytotoxic effects on biomolecules, including proteins, lipids and DNA. In this context, some *in vivo* studies have demonstrated that several oxidative markers are affected by following ethanol chronic administration. For example, glutathione (GSH) levels and GSH peroxidase activity have been observed to be diminished in the sciatic nerves of ethanol-fed rats compared to pair-fed rats; on the contrary, the amount of the lipid peroxidation product malondialdehyde increased in the same tissue (Bosch-Morell et al., 1998). More recently, Tiwari et al. (Tiwari et al., 2009, 2011) have confirmed significant increased levels of lipid peroxidation and marked decrease in GSH, superoxide dismutase and catalase activities in the sciatic nerve of rats which were given alcohol for 10 weeks. Alcohol has been also found to enhanced production of hydrogen peroxide and ^•^OH like species (Dicker and Cederbaum, 1992). As mentioned above, ROS overproduction may lead to sensitization of dorsal horn cells, activation of spinal glial cells and inflammatory response, which ultimately activate PKC and nuclear factor Kβ translocation, MEK/ERK signaling and apoptosis.

### Oxidative stress dependent TRP channels and pain

It is well known that ROS and RNS are produced in several physiological functions, such as mitochondrial and cytochromes P450 activities. These free radicals are scavenged by enzymatic and non-enzymatic antioxidants. At 2002, two different groups from Kyoto-Japan (Bautista et al., 2005) and Aachen-Germany (Kistner et al., 2016) reported activation of a TRP channel namely LTRPC2 (former name of TRPM2) by RNS and ROS. Today, 9 TRP channels (TRPA1, TRPC5, TRPM2, TRPM4, TRPM7, TRPV1, TRPV2, TRPV3, and TRPV4) are demonstrated to be activated by oxidative stress (Mori et al., 2016). Expression levels of these channels are very different in tissues and cells. For example, it has been observed that the expression levels of four TRP channels (TRPA1, TRPM2, TRPV1 and TRPV4) are high in neurons related to nociception. Hence, this section is focused on these four TRP channels.

### TRPA1

Cysteine is a sulfur-containing amino acid in humans. Cysteine, as a source of thiol redox system, acts also as the main source of different antioxidants such as GSH, glutathione peroxidase, N acetyl cysteine (NAC) and α-lipoic acid. Hence, the cysteine groups are main target for ROS and RNS (Sen and Packer, 2000; Naziroǧlu, 2007).

The TRPA1 channels are activated by different stimuli including chemicals (mustard oil and cinnamaldehyde) and cold body temperature (≤17°C). In addition, TRPA1 is also an oxidative stress-sensitive Ca^2+^-permeable channel. Therefore, activation of TRPA1 in neurons by oxidative stress such as H_2_O_2_ was reported (Materazzi et al., 2012; Bai and Lipski, 2013; Toda et al., 2016). Furthermore, the TRPA1 channel is activated by depletion of intracellular GSH, although its activation in the DRG neurons was inhibited by antioxidants of thiol redox system, such as GSH and selenium (Materazzi et al., 2012; Özdemir et al., 2016; Kahya et al., 2017) (Table 1).

**Table 1:** Role of reactive oxygen species (ROS) and reactive nitrogen species (RNS) on the activation of transient receptor potential channels (TRPA1, TRPM2, TRPV1, and TRPV4) in the peripheral neuron.

**Channel**	**ROS/RNS**	**Material**	**Value**	**Reference**
TRPA1	H_2_O_2_,	Rat DRG	Protective role of GSH on peripheral pain through inhibition of cysteine oxidation.	Bosch-Morell et al., 1998; Materazzi et al., 2012
TRPA1	Nitric oxide	Rat DRG	Protective role of dithiothreitol, cysteine and GSH on peripheral pain through inhibition of nitric oxide production.	Naziroǧlu, 2007; Tiwari et al., 2011
TRPM2	ADP-ribose and H_2_O_2_	Rat DRG	NADPH oxidase dependent activation of TRPM2	Naziroglu and Braidy, 2017
TRPM2	ADP-ribose and H_2_O_2_	Rat DRG	Protective role of GSH and NAC on peripheral pain and channel activation through inhibition of oxide stress.	Lee et al., 2017; Miyamoto et al., 2017; Naziroglu and Braidy, 2017
TRPV1	H_2_O_2_ and nerve growth factor	Rat DRG	Protective role of GSH, selenium and NAC on hyperalgesia and channel activation through inhibition of oxide stress.	Mei et al., 2006; Naziroǧlu et al., 2011b; Kahya et al., 2017
TRPV1	H_2_O_2_	Mice DRG	Oxidative stress-induced inflammatory hyperalgesia	Sözbir and Naziroǧlu, 2017
TRPV1	RNS and NADPH oxidase	Mice and Rat DRG	Oxidative stress-induced inflammatory hyperalgesia and channel activation	Caterina et al., 1997; Naziroglu and Braidy, 2017
TRPV4	H_2_O_2_ and ROS	Rat DRG	Protective role of GSH on peripheral pain through inhibition of cysteine oxidation	Dina et al., 2008; Materazzi et al., 2012
TRPV4	ROS	Rat DRG	Protective role of TRPV4 blockers on mechanical allodynia and oxidative stress	Materazzi et al., 2012

It is well known that increase of intracellular ROS, RNS and Ca^2+^ has main roles in etiology of pain processes (Kallenborn-Gerhardt et al., 2012; Ogawa et al., 2016). As it was mentioned above, the TRPA1 and TRPV4 channels are activated by different stimuli, including oxidative stress (Bai and Lipski, 2013). Involvement of TRPA1 channels in the etiology of pain processes has not been fully clarified yet, although there are some reports on TRPA1 activation-induced pain processes such as diabetic peripheral pain (Andersson et al., 2015; Jardín et al., 2017; Kahya et al., 2017) and SCI-induced pain (Park et al., 2015; Klafke et al., 2016) and chemotherapeutic agent-induced pain (Naziroglu and Braidy, 2017) through excessive ROS and RNS production in the rodents. In addition, it was reported in DRG neurons of wild type mice and TRPA1 knockout mice that activation of TRPV1 by chemotherapeutic agents induced excessive ROS production and mechanical allodynia. However, TRPA1 and TRPV4 antagonist treatments induced decrease on the allodynia and oxidative stress in the mice (Materazzi et al., 2012). In a previous study, the same group did not observe Ca^2+^ response effects induced by exposure to chemotherapeutic agents in cultured mouse DRG and Chinese hamster ovary (CHO) cell line (Nassini et al., 2011), although chemotherapeutic agent evoked an antioxidant GSH-sensitive Ca^2+^ response in the CHO cell line and DRG neuron. Results of a study indicated that chemotherapeutic agents-induced oxidative stress caused TRPA1 activation instead of direct channel targeting (Nassini et al., 2011). The report was confirmed by a recent study; thus, it was observed that chemotherapeutic agent-induced increase of TRPA1 expression, cell death and neuropathic pain in mice DRG was reduced by aluminum and GSH treatment (Lee et al., 2017). TRPA1 activator role of hydrogen sulfide through nitric oxide production was recently reported in DRG neuron too (Miyamoto et al., 2017), as shown in Table 1.

There is a synergic interaction between TRPA1 and TRPV1 on channel activation mechanisms in DRG, because TRPA1 is co-localized with 30–50% TRPV1 expressing neurons in rat and human DRG (Bautista et al., 2005). Therefore, the sensitization ratio of TRPA1 is affected by several factors, including oxidative stress and TRPV1 blocker (Kistner et al., 2016; Ogawa et al., 2016). On the subject, increased sensitization of human TRPA1 in DRG neuron was reported by inflammation and oxidative stress, although the increased sensitization in the neuron is decreased by antioxidant NAC and capsazepine (Kistner et al., 2016).

### TRPM2

Another member of TRP superfamily is TRPM2. The enzyme (ADP ribose) pyrophosphatase in the C-terminal domain of TRPM2 contains is sensitive to ROS and RNS (Naziroǧlu, 2007). TRPM2 channel in transfected cell lines is gated by extracellular and intracellular ROS, possibly by interacting with the ADP ribose pyrophosphatase enzyme in the tail of the protein C domain (Perraud et al., 2001; Hara et al., 2002; Wehage et al., 2002). Later, TRPM2 activator role of oxidative stress from ADPR was reported in transfected cells by single channel patch-clamp experiments (Naziroglu and Lückhoff, 2008). Presence of TRPM2 function in DRG neuron was firstly reported in 2011 (Naziroǧlu et al., 2011a). It was highlighted that the excessive ROS production through activation of NADPH oxidase contributes to sensitization in DRG neuron for persistent pain induction (Kallenborn-Gerhardt et al., 2012). Result of a more recent study indicated involvement of NADPH oxidase on TRPM2 channel activation in DRG neuron (Naziroǧlu, 2017) (Table 1). In addition to the TRPA1 channel, involvement of cysteine groups on the activation of TRPM2 channels in transfected human embryonic kidney (HEK-293) cells was reported (Mei et al., 2006). Then, protective roles of GSH and NAC as members of thiol redox system on TRPM2 channel and peripheral pain inhibition in DRG neuron were also reported (Naziroǧlu et al., 2011b; Özgül and Naziroǧlu, 2012; Sözbir and Naziroǧlu, 2017) (Table 1). It seems that members of thiol redox system have important roles on inhibition of oxidative stress-dependent TPM2 channel activation and peripheral pain in rodents.

### TRPV1

A subfamily of TRP superfamily is vanilloid family. TRPV1 is a member of the vanilloid subfamily. The channel was firstly expressed in rats through activation of high temperature and pungent hot chili pepper component (capsaicin) in mice DRG neuron (Caterina et al., 1997). The channel can also be activated by different stimuli including low pH (<5.9), high temperature (>43°C) and oxidative stress leading to the perception of pain, and oxidative injury (Yoshida et al., 2006). As most of cation channel protein, TRPV1 channel protein contains six transmembrane domains. Similar to TRPA1 (Takahashi et al., 2011) and TRPM2 (Mei et al., 2006) membrane structure, oxidative alterations of multiple Cys residues in different cells are involved in this mode of TRPV1 activation by modifying (Yoshida et al., 2006; Chuang and Lin, 2009) and disulfide bond formation (Wang and Chuang, 2011). Therefore, the TRPV1 is activated in rat DRG neuron by depletion of intracellular GSH (Naziroglu et al., 2013), although hyperalgesia and the TRPV1 channel were inhibited in the DRG neurons of rats by treatment of thiol redox cycle members such as GSH, selenium and NAC (Khodorova et al., 2013; Naziroglu et al., 2013; Kahya et al., 2017) (Table 1).

Excessive ROS are produced in physiological functions such as mitochondrial function and phagocytic activity. During the killing bacteria and virus, ROS are used in the anti-inflammatory cells such as macrophages microphages and microglia. Therefore, there is a direct relationship between increased levels of ROS and inflammatory hyperalgesia (Oehler et al., 2017). Interactions between TRPV1 and long sustained thermal hypersensitivity in oxidative stress-induced inflammatory hyperalgesia of mouse hind paw were reported (Keeble et al., 2009). Therefore, there is a direct role of oxidative stress through activation of TRPV1 on hyperalgesia in DRG neuron of wild type and Nox1 deficient mice (Ibi et al., 2008) (Table 1). Niflumic acid is also a TRPV1 channel antagonist and it was reported that peripheral neuropathy by suppressing excessive ROS, RNS, inflammatory cytokine production and TRPV1 activation in neuropathic pain-induced rats were recovered by the niflumic acid treatment (Marwaha et al., 2016).

### TRPV4

A member of TRP superfamily is TRPV4 and it was firstly described with mammalian osmo-transducer property (Liedtke et al., 2000). The channel is also activated by phorbol esters, low pH, citrate, arachidonic acid, exogenous chemicals (bisandrographolide A) and heat (24°C ≥) (Güler et al., 2002; Yoshida et al., 2006). In addition to the stimulators, activation of TRPV4 in neurons by oxidative stress such as H_2_O_2_ was reported (Materazzi et al., 2012; Bai and Lipski, 2013), although its activation in the DRG neurons was inhibited by GSH (Materazzi et al., 2012) (Table 1). It was also reported in DRG neurons of wild type and TRPV4 knockout mice that activation of TRPV4 by paclitaxel induced mechanical allodynia and excessive ROS production, although the allodynia and oxidative stress was partially decreased by the TRPV4 antagonist treatment (Materazzi et al., 2012) (Table 1).

### Role of other factors on TRP channels and pain

In addition to the oxidative stress, several other factors play a role in the induction of CIPN with/without TRP channel activation, including glutamate receptors, neuropeptides, PKC and inflammation. Although their role in CIPN has been known for a long time, there are limited reports about the interaction between these factors and TRP channels in the literature. In this section, some brief information is given about other main factors and TRP channels activation related to pain induction in experimental animals.

1. Glutamate receptors and neuropeptides: Many types of ionotropic glutamate receptors were reported in literature. Presence of three types of ligand-gated glutamate ion channel receptors such as N-methyl-D-aspartate, α-amino-3-hydroxy-5-methyl-4-isoxazolepropionic acid, and kainite receptors has been known for a long time. Their involvement in CIPN was also investigated by numerous papers (Wu and Zhuo, 2009; Vécsei et al., 2015). On the other hand, substance P and calcitonin-gene-related-neuropeptide are well known neuropeptides in the induction of peripheral neuropathy and nociception. Their implication in CIPN through the overload of Ca^2+^ entry has been demonstrated (Hill and Oliver, 2007; Nassini et al., 2014). In addition, recent studies have pointed out the involvement of neuropeptides on TRP channel activation, such as TRPA1, TRPM8 and TRPV1 (Nassini et al., 2014; Chukyo et al., 2018). For example, it was observed that substance P was potentiated by the sensitization of TRPV1 (Marwaha et al., 2016).
2. PKC enzyme: Apart from the involvement of PKC on the induction of oxidative stress in neurons, both human and animal studies have linked this enzyme to several pain types through excessive Ca^2+^ entry and cation channel activation (Carozzi et al., 2015; Kumar et al., 2017). In this sense, the involvement of PKC in experimental models of CIPN was also evidenced by several papers, although mechanical hyperalgesia was decreased by the treatment with PKC inhibitors such as hypericin and calphostin C (Norcini et al., 2009). Sensitivities of nociceptive neurons to heat stimulation and TRPV1 activation were increased by the activation of PKC and inflammatory mediators (Khasar et al., 1999; Gao et al., 2016). There is also direct relationship between PKC and onset of mechanical hyperalgesia (Khasar et al., 1999; Hucho et al., 2006). It was reported that paclitaxel-induced mechanical hypersensitivity were increased in the DRG of mice by up-regulation of PKC (Dutra et al., 2015). Moreover, antioxidants such as quercetin were able to inhibit TRPV1 and PKC activations in a study of paclitaxel-induced peripheral neuropathy (Gao et al., 2016).
3. Inflammation: Brain and spinal glia neurons have a main role on homeostasis in central nervous system and peripheral nervous system. The glial cells have been shown to contribute to the development of chronic pain as results of surgery, inflammation, and SCI. Therefore, reduction of NP was observed by treatment of the glial activity (Carozzi et al., 2015). Involvement of oxidative stress-induced inflammation was reflected in other parts of this review (see “Chemotherapy-induced peripheral neuropathy” and “Spinal cord injury” sections). The increased glial neuron-induced inflammation induces pain through TRP channel activation in DRG and spinal cord neurons. For example, TRPV1 and TRPA1 are activated by several stimuli related with traumatic brain injury, including mechanical shear stress, leading to the release of substance P and inflammation (Corrigan et al., 2016). Enhanced expression and spinal inflammation-induced sensitization of TRPV1 and streptozotocin-induced thermal hyperalgesia and neuropathy were reported in rats (Bishnoi et al., 2011). Involvement of TRPV1 in the activation of spinal glia in mice with nociceptive, inflammatory and neuropathic pain was also reported (Chen et al., 2009).

### Antioxidant supplementation

In general, scientific evidence reinforces the future use of antioxidant supplementation in several pathological conditions. Taking in account previous works of our research group (Carrasco et al., 2013, 2014a,b) and other authors, both preventive and therapeutic uses of antioxidants have been reported to reduce not only oxidative stress related parameters but also inflammatory response and pain in several diseases. As mentioned earlier, it has been demonstrated that current drugs used in the management of different kinds of NP are ineffective and usually not safe for the patient. For this reason, antioxidant supplementation might be an alternative to take in account in clinical practice.

As an illustration, oral administration of molecular hydrogen may have therapeutic potential for the management of NP. Unlike other antioxidants, hydrogen reaches target organs easily, where selectively neutralizes ^•^OH, and does not accumulate in living cells nor produce noxious metabolites (Kawaguchi et al., 2014). Naringenin, an abundant flavanone in citrus fruits (Kaulaskar et al., 2012) and genistein, a natural phytoestrogen from soybean (Valsecchi et al., 2008), have been exhibited analgesic, antioxidant and immunoregulatory properties in sciatic nerve injury models. α-lipoic acid treatment (600 mg/day) has also demonstrated to improve neuropathic symptoms (pain, burning, paresthesia, and numbness) and deficits in patients with NP (Tesfaye, 2011).

Concerning antioxidant supplementation in CINP, natural antioxidants such as curcumin (Al Moundhri et al., 2013), silibinin, α-tocopherol (Kerckhove et al., 2017), rutin and quercetin (Azevedo et al., 2013) have exhibited antinociceptive effects in oxaliplatin induced-CINP. For example, administration of the flavonoids rutin and quercetin has shown to diminish oxidative phenomena including lipid peroxidation, nitrosylation and iNOS expression, as well as pain symptomatology (thermal and mechanical allodynia) in treated mice compared to non-treated ones (Azevedo et al., 2013). Likewise, the thiol compound NAC has demonstrated to exert a beneficial effect in the treatment of oxaliplatin induced-CINP, significantly reducing inflammatory response (TNF-α, IL-1β and IL-6) in the neuroblastoma cell line SH-SY5Y; additionally, researchers also observed that NAC prevented apoptosis by inhibition of P2X7 receptor activation by blocking ROS production and caspase-3 activation. Interestingly, in this study NAC exhibited the highest preventive effect compared to other drugs also tested such as ibuprofen and acetaminophen (Massicot et al., 2013). On the other hand, prophylactic treatment with acetyl-L-carnitine has demonstrated to prevent paclitaxel-, oxaliplatin- and bortezomib-induced mitochondrial dysfunction and pain (Zheng et al., 2012). In spite of the promising results, most clinical studies related to the use of antioxidants as chemotherapy adjuncts did not report on their impact on anticancer efficacy; hence, as Han et al. (Han and Smith, 2013) highlighted this is an important question which should be examined in greater depth. In this sense, previous *in vitro* studies performed by our research group have shown that co-administration of conventional chemotherapeutic agents with antioxidants such as melatonin enhances chemotherapeutic-induced cytotoxicity and apoptosis in different cancer cell lines (Uguz et al., 2012; Pariente et al., 2016, 2017a,b). In addition, since some studies have also recorded failure in antioxidant supplementation efficacy, optimal design of clinical trials in terms of targeted delivery of antioxidants, clinical pathology and concentration dependent dosage schedule is needed to go ahead in the knowledge and future application of this kind of treatment in CIPN (Kamat et al., 2008). Finally, as Areti et al. (2014) point out monitoring oxidative stress related-parameters (i.e., levels of malondialdehyde, GSH, superoxide dismutase and activities of mitochondrial enzymes such as citrate synthase and ATP synthase) during the course of CIPN could be helpful in clinical practice.

### Future consideration

Taking in account the scientific evidence summarized in this review, NP is a complex network of several molecular processes, including nitro-oxidative stress, immune response, and TRP channels activation, among others. Noteworthy, NP seems to be not only promoted by direct injury to neurons but also by TRP channels mediating damage in the surrounding tissue. However, the way how these actors and other factors (e.g., sodium channels, acid-sensing ion channels and synaptic receptors) are interconnected leading to noxious symptomatology remains to be unresolved.

Since 1990s, with the discovery of TRP channels, our understanding about nociception has changed. Most nociceptive TRP channels are predominantly expressed in peripheral sensory neurons, but there is also a significant expression in the central nervous system and other tissue and cell types (i.e., keratinocytes, vascular endothelial cells, bladder epithelial cells, fibroblasts and human dental pulp) (Mickle et al., 2016). Thus, we are still far from a complete understanding of the biology of nociception and its applicability in clinical practice. Current evidence points out the possibility that multiple nociceptive TRP channels are activated during pathological conditions, including the nine oxidative sensitive-TRP channels known until today. Although there are several reports on four oxidative sensitive-TRP channels reviewed in this paper, there is no report linking the remaining oxidative sensitive-TRP channels (TRPM4, TRPM7, and TRPC5) and pain in the peripheral neurons. In this sense, contradictory results have been obtained about the expression of certain TRP channels in DRG neurons, such as TRPV4; in addition, TRPV2-6 and TRPM3 expression at this level is unknown. Furthermore, information about some important aspects related to TRP channels, including their location, trafficking, functionality and overlapping in neurons and other kind of cells, both in physiological and pathophysiological states, is lacking. Besides their neuronal/plasma membrane location, it has been observed that a significant fraction of TRP channels is also present in organelles membranes which may be translocated as required by cells exposed to injury/inflammation (Mickle et al., 2016). Finally, it should be noted that some populations consume large amounts of capsaicin, which is well known to activate TRPV1 channels and to induce overload Ca^2+^ entry in hippocampal and DRG neurons (Kahya et al., 2017). In recent papers, we have reported the involvement of TRPV1 channels in the induction of epilepsy (Naziroǧlu, 2015; Naziroglu and Övey, 2015). In this sense, some Turkish populations have been traditionally consuming high amounts of hot chili pepper (capsaicin) in food and it has been observed a high incidence of epilepsy in these areas (unpublished data). Thus, similar possible relationship between high amount hot chili pepper consumption and several peripheral pain inductions should be investigated by future studies. Furthermore, the relevance of TRPs in NP will remain elusive until experimental studies (including knockdown or knockout animal models) demonstrate that an increase in TRP activity by exogenous TRP activators produces NP.

In any case, TRP channels are now presented as attractive targets for the development of new-generation analgesics. Until today, there are many small molecule blockers of TRPV1 (AZD1386) (Clinical Trials, 2012), TRPA1 (GRC-17536) (Clinical Trials, 2014) and TRPV3 (SAR292833) (Clinical Trials, 2016), apart from topical TRPV1 agonists, such as zucapsaicin (Clinical Trials, 2011) and NGX 4010 (Mou et al., 2014), that have been tested in clinical trials of several NP conditions; to the best of our knowledge, NGX 4010 is the only compound that has been launched for clinical use in human post-herpetic neuralgia-NP conditions. Concerning to cancer pain, a phase I clinical trial is being carried out to determine the efficacy of periganglionic/intrathecal administration of the potent TRPV1 agonist resiniferatoxin in advanced cancer patients with bone pain (Clinical Trials, 2017). However, it is suspected that other nociceptive TRP channels may be involved in cancer pain. Therefore, this and other questions, such as efficacy and site of action of drugs targeting nociceptive TRP channels, will need to be answered in the next years.

## Conclusions

NP is an underestimated socioeconomic health problem affecting millions of people worldwide, which incidence may increase in the next years due to chronification of several diseases such as cancer and diabetes. Nitro-oxidative stress and inflammatory response, with the consequent activation of TRP channels, seem to play a major role in the beginning and development of NP. Hence, it is now urgent to discover new, effective and safe strategies to prevent and/or treat this hardly bearable condition. Recent discoveries in different biomedical fields point out the need to change paradigms about pharmacological management of diseases. From our point of view, therapeutic options must not only be directed to reach a molecular target, which ultimately would represent a fixed picture of the disease, but also to restore physiological global context in terms of nitro-oxidative stress and inflammatory response, just as the antioxidant treatment seems to act. Furthermore, increasing aging population and chronic diseases prevalence demand the development and implementation of antioxidant therapies in clinical practice. But it must not be forgotten that possible prevention of several diseases following a varied and balanced diet, as well as other healthy habits, is a reality nowadays. Health care institutions, clinicians and general population must be aware of the importance of nutrition, as source of natural antioxidants, in our physical and mental state, as much in health as in illness.

## Author contributions

CC and MN wrote the review. AR and JP critically revised the work and approved its version to be submitted.

### Conflict of interest statement

The authors declare that the research was conducted in the absence of any commercial or financial relationships that could be construed as a potential conflict of interest.

## Abbreviations

APN: Alcoholic peripheral neuropathy
CIPN: chemotherapy-induced peripheral neuropathy
DRG: dorsal root ganglion
ETC: mitochondrial electron transport chain
GSH: glutathione
NAC: N acetyl cysteine
NP: neuropathic pain
PARP-: poly (ADP-ribose) polymerase-1
PDN: peripheral diabetic neuropathy
PKC: protein kinase C
ROS: reactive oxygen species
RNS: reactive nitrogen species
SCI: spinal cord injury
TRP: transient receptor potential
TRPA1: transient receptor potential ankyrin 1
TRPM2: transient receptor potential melastatin 2
TRPV1: transient receptor potential vanilloid 1
TRPV4: transient receptor potential vanilloid 4.
